# Spontaneous fistulization of walled‐off pancreatic necrosis into the colon

**DOI:** 10.1002/ccr3.2946

**Published:** 2020-05-19

**Authors:** Tanveer Khan, Narendra Pandit

**Affiliations:** ^1^ Surgical Gastroenterology Division Department of Surgery B P Koirala Institute of Health Sciences (BPKIHS) Dharan Nepal

**Keywords:** colon, fistula, necrosis, pancreatitis

## Abstract

Spontaneous fistulization of the pancreatic necrosis into the colon is rare. It should be kept as differentials in the presence of massive air in the WOPN. Sometimes, simple bedside X‐ray abdomen may clinch the diagnosis in the presence of large radiolucent air‐fluid level with a peripheral displaced bowel loops.

## Graphical abstract

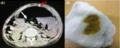

A 17‐year‐old girl presented with 2‐month history of epigastric pain. She also complained of an abdominal lump for the past few days. She had been under treatment for severe acute pancreatitis at an outside hospital. On examination, she was ill‐looking, febrile, tachycardic with a normal blood pressure. An abdominal examination revealed central abdominal distension with a localized peritonism. X‐ray abdomen revealed a large radiolucent opacity in central part of the abdomen displacing bowel loops at the periphery, with a large volume air‐fluid level (Figure 1). On further confirmation with computed tomography abdomen, it showed a walled‐off pancreatic necrosis (WOPN) with a massive air density (Figure 2). Image‐guided percutaneous catheter drain was placed in the WOPN, which initially drained air with feculent content (Figure 2) and then the pancreatic necrosum suggesting spontaneous colonic fistulization.

**Figure 1:**
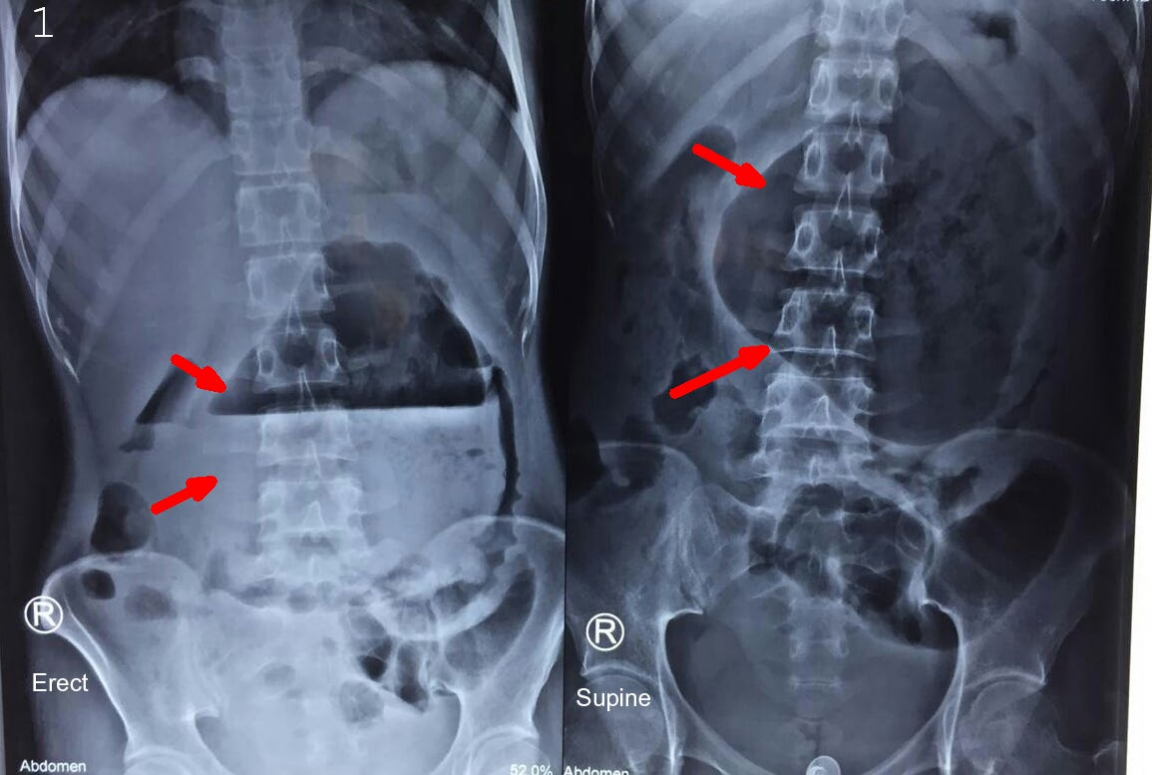
X‐ray abdomen showing large radiolucent opacity in the central part of the abdomen (*red arrow*) displacing the bowel loops at the periphery, with an air‐fluid level

**Figure 2:**
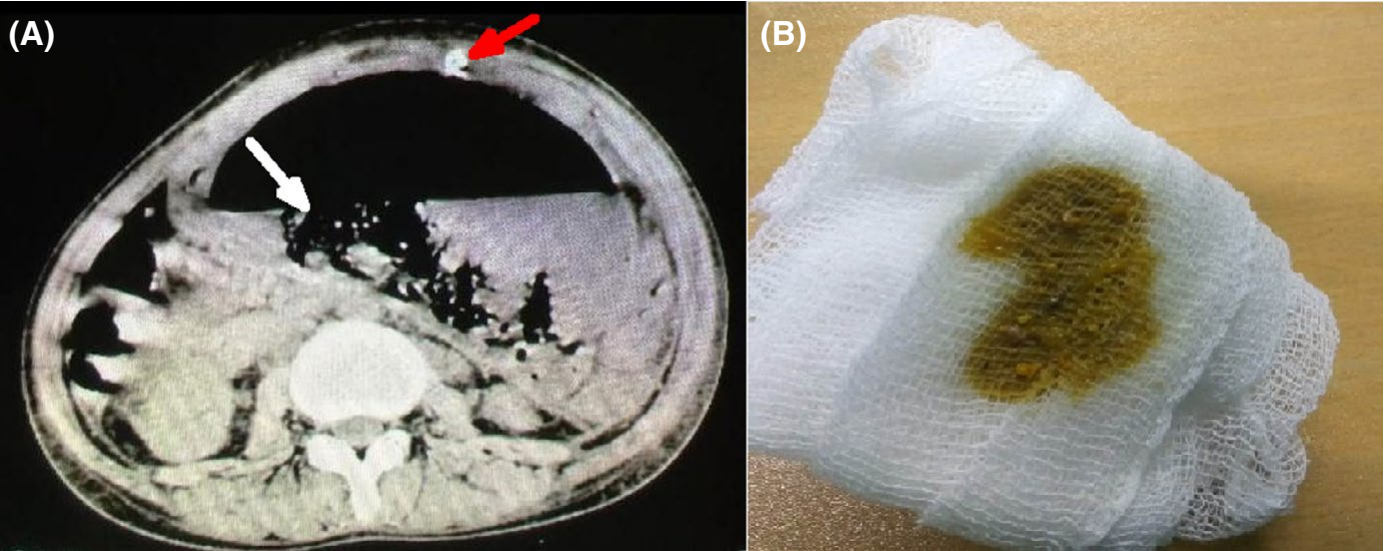
A, Computed tomography (CT) abdomen showing massive air in the pancreatic necrosis (*white arrow*). Note the pigtail catheter tube in the abdominal wall (*red arrow*). B, The feculent content drained by the pigtail catheter suggesting colonic fistula

Fistulization of the gastrointestinal tract (GI) is an uncommon complication of necrotizing pancreatitis. It generally occurs following interventions or rarely spontaneously.^1^ The presence of air in WOPN suggests infection, but when large‐volume air is seen, it suggests fistulization.^2^ Rarely, due to the massive air, it can present with an abdominal lump, where the diagnosis can be clinched from the simple abdominal X‐ray, as seen in our case.

## CONFLICT OF INTEREST

None.

## AUTHOR CONTRIBUTIONS

TK: conceptualized the study and collected the data. NP: wrote the paper, coordinated the study, approved, and drafted the final manuscript.
